# Implementation and delivery of group consultations for young people with diabetes in socioeconomically deprived, ethnically diverse settings

**DOI:** 10.1186/s12916-022-02654-0

**Published:** 2022-11-25

**Authors:** Chrysanthi Papoutsi, Dougal Hargreaves, Ann Hagell, Natalia Hounsome, Helen Skirrow, Koteshwara Muralidhara, Grainne Colligan, Anne Ferrey, Shanti Vijayaraghavan, Trish Greenhalgh, Sarah Finer

**Affiliations:** 1 Nuffield Department of Primary Care Health Sciences, University of Oxford, Radcliffe Observatory Quarter, Woodstock Road, Oxford, OX2 6GG UK; 2 School of Public Health, Imperial College London, London, UK; 3 Association for Young People’s Health, London, UK; 4 Brighton and Sussex Medical School, Brighton, UK; 5 London Northwest University Healthcare NHS Trust, London, UK; 6 Centre for Primary Care and Public Health, Blizard Institute, Barts and The London School of Medicine and Dentistry, Queen Mary University of London, London, UK; 7 Barts Health NHS Trust, London, UK

**Keywords:** Group consultations, Shared medical appointments, Diabetes, Young people, Complexity

## Abstract

**Background:**

Young people with diabetes experience poor clinical and psychosocial outcomes, and consider the health service ill-equipped in meeting their needs. Improvements, including alternative consulting approaches, are required to improve care quality and patient engagement. We examined how group-based, outpatient diabetes consultations might be delivered to support young people (16–25 years old) in socio-economically deprived, ethnically diverse settings.

**Methods:**

This multi-method, comparative study recruited a total of 135 young people with diabetes across two implementation and two comparison sites (2017–2019). Informed by a ‘researcher-in-residence’ approach and complexity theory, we used a combination of methods: (a) 31 qualitative interviews with young people and staff and ethnographic observation in group and individual clinics, (b) quantitative analysis of sociodemographic, clinical, service use, and patient enablement data, and (c) micro-costing analysis.

**Results:**

Implementation sites delivered 29 group consultations in total. Overall mean attendance per session was low, but a core group of young people attended repeatedly. They reported feeling better understood and supported, gaining new learning from peers and clinicians, and being better prepared to normalise diabetes self-care. Yet, there were also instances where peer comparison proved difficult to manage. Group consultations challenged deeply embedded ways of thinking about care provision and required staff to work flexibly to achieve local tailoring, sustain continuity, and safely manage complex interdependencies with other care processes. Set-up and delivery were time-consuming and required in-depth clinical and relational knowledge of patients. Facilitation by an experienced youth worker was instrumental. There was indication that economic value could derive from preventing at least one unscheduled consultation annually.

**Conclusions:**

Group consulting can provide added value when tailored to meet local needs rather than following standardised approaches. This study illustrates the importance of adaptive capability and self-organisation when integrating new models of care, with young people as active partners in shaping service provision.

**Trial registration:**

ISRCTN reference 27989430.

**Supplementary Information:**

The online version contains supplementary material available at 10.1186/s12916-022-02654-0.

## Background

Improving quality and efficiency in diabetes care is necessary in the context of rising global prevalence [1]. Despite recognised standards that identify priority areas for improvement and monitoring [2, 3], gaps in service provision remain and continue to lead to poor outcomes [4]. There is an urgent need to examine how diabetes care can be structured differently to meet rising demand and support good self-care practices.

Young people are particularly affected by suboptimal care which does not address their complex physical, emotional, and developmental needs [5, 6]. In England and Wales, only half of young patients with type 1 diabetes receive all recommended health checks and their glycaemic markers remain consistently above target levels set by the National Institute for Health and Care Excellence (NICE), according to the 2019/20 paediatric audit [7]. Equally, in the USA, only a minority of young people achieve recommended HbA1c goals [8]. Health disparities mean those from socio-economically deprived areas experience worse outcomes and complications [9, 10]. Young people face significant barriers engaging with the service including diabetes-related psychological distress and fear of complications [11], with studies reporting poor experiences and deterioration in the transition to adult care [12, 13] and lack of life-stage oriented therapeutic relationships [14, 15].

Novel approaches, such as group consultations (or ‘shared medical appointments’), have been employed to address current shortcomings in diabetes care delivery [16–21]. Group consultations bring together clinical experience and patient expertise to provide an alternative means of service provision, where small groups of patients attend jointly for their clinical consultations and at the same time have the opportunity to share with peers [22]. Extending beyond patient education, self-management, and peer support programmes (long established in diabetes care), it has been suggested that group consultations contribute towards improvement of clinical and patient-reported outcomes [21, 23]. This model of care (implemented differently in different settings and for different purposes, e.g. see [24]) has also been trialled in general practice and across a range of other conditions including asthma, hypertension and prenatal care [25–28].

To better understand how (and whether) this new model of care could deliver intended outcomes for young people with diabetes in socio-economically deprived, ethnically diverse settings, we implemented and evaluated a group consultations programme in 2 UK hospitals between 2017 and 2019, following a co-production approach [29, 30]. The multi-method evaluation focused on the following research questions:

- What are the key challenges in implementation and delivery of group consultations to improve outpatient diabetes care?
- How do young people and staff experience this new model of care, compared to one-to-one outpatient consultations?
- What differences are there between young people attending and not attending group clinics, from a sociodemographic, patient enablement, service use, and clinical perspective?
- What are the costs of setting up and delivering group consultations?

## Methods

A ‘researcher-in-residence’ [31] was involved in all aspects of the group consultations programme in the 2 implementation sites (2017–2019). At the time, she held a postdoctoral university role and contributed to the study from a social science (including on healthcare evaluation) rather than clinical perspective. Having completed a review of relevant literature ahead of implementation [22], she supported the translation of empirical evidence in practice, worked closely with frontline clinical and implementation teams, and fed back qualitative findings.

Table 1 sets out key characteristics of the 2 implementation sites and group consultations delivered. The group consultations programme was co-designed with experts in supporting young people’s health who led dedicated workshops and individual sessions with patients, clinical and non-clinical staff, a commissioner, and representatives from primary care and the voluntary sector. The programme was further refined through iterative co-production in the context of service provision (e.g. feedback discussions at the end of group consultations) to allow adaptation over time (for more details, see full study report [29]).

**Table 1:** Summary of implementation site characteristics

	Implementation site A (September 2017–May 2019)	Implementation site B (September 2018–September 2019)
**Setting**	East London, 72% ethnically minoritised groups, 52% childhood poverty rate	Northwest London, 65% ethnically minoritised groups, 43% childhood poverty rate
**Approximate size of young adult population in standard diabetes care**	200 young adults with diabetes aged 16–25	75 young adults with diabetes aged 16–25
**Group consultations delivered**	23 group consultations	6 group consultations
**Standard diabetes care**	Monthly multidisciplinary clinic and weekly nurse clinic, with virtual option and mobile phone access	Multidisciplinary clinic twice per month, with daily walk-in clinics and mobile phone access. Care led by adult diabetes team, but with close work with the paediatric team post-transition
**Staffing**	Consultant diabetologist, diabetes specialist nurse, dietitian, psychologist, and youth worker.	Consultant diabetologist, diabetes specialist nurse, dietician, with input from psychologist
**Other relevant features**	Recent service improvement work, e.g. offering peer support groups, video consultations	Recent service improvements including delivery of structured education (TEAM T1) for young adults

Qualitative methods included the following: (a) ethnographic observation in group consultations and individual diabetes appointments, as well as relevant implementation work, such as co-ordination meetings, facilitation training, co-design sessions, staff preparation ahead of group consultations, and other encounters (approx. 120 h); (b) 31 semi-structured interviews with young people with diabetes, diabetes consultants, nurses, and other clinical and non-clinical staff (this includes one joint interview with two young sisters and two repeat interviews with a staff member to understand changes over time); and (c) review of relevant documents and materials (Table 2). Interviews lasted 30–110 min with the majority taking place in the clinics; 7 participants preferred to speak on the phone. Interviews and group consultations (including short 10–15’ feedback discussions with young people facilitated by the researcher or the youth worker at the end of group consultations) were audio-recorded with consent and professionally transcribed (Table 3).

**Table 2:** Data collection and summary of data sources

	Methods	Data overview
**Qualitative data collection**	A. Interviews with young people with diabetes (*n* = 19) - 9 female/10 male - 18–25 years old - Varied ethnic backgrounds - 17 living with type 1 diabetes, 2 with type 2 - 4 had attended 7-–10 clinics, 9 had attended 3–6 clinics, and 6 had attended 0–2 clinics (one of whom withdrew from the research study after one group clinic, and another consented but never attended) B. Interviews with staff participants (*n* = 11) - 3 diabetes consultants - 3 diabetes specialist nurses - 1 youth worker - 1 research officer - 1 dietician - 1 psychologist - 1 sexual health advisor	a. Interview transcripts b. Field notes
**Ethnographic observation**	A. Observation in clinical encounters (~70 h) - 29 audio-recorded group clinics in 2 hospital sites, including feedback discussions with participants to support co-production - 15 individual diabetes consultant and nurse appointments B. Observation in non-clinical encounters in the context of the implementation study (~50 h) - Co-design sessions, implementation and advisory group meetings, facilitation training, preparation for group clinics, other informal observations (observations involved clinicians implementing group consultations, other members of the implementation team and diabetes service in the 2 hospitals, advisory board members and service and patient participants in co-design)	a. Group clinic transcripts and field notes b. Feedback discussion transcripts and field notes c. Field notes from individual appointments d. Materials used in group clinics (e.g. flip charts, icebreakers, handouts, etc.) e. Field notes and documents used in meetings/other encounters
**Quantitative data collection**	A. Structured data collection proformas from clinical records B. Patient-reported instruments - Patient Enablement Instrument (PEI) - Problem Areas in Diabetes (PAiD) - Patient questionnaires C. Bespoke costing templates completed by care teams	a. Clinical data (diabetes type and relevant biomarkers, age at diagnosis, technology use, previous diabetes education attendance) b. Service use data within the last year (diabetes appointments, emergency attendances (diabetes-related), inpatient admissions (diabetes-related), primary care consultations) c. Patient enablement, psychological outcomes and diabetes distress d. Sociodemographic data (age, sex, multiple deprivation index, ethnicity, English as first language, education, employment status) e. Economic data on estimate use of resources and cost of group clinics

**Table 3:** Group clinic structure

Preparing for the group clinic	Invitations to group clinic via usual care processes, with additional telephone/SMS communication from youth worker
	Topic/theme for the group clinic confirmed and young adults notified in SMS invitation
	Invitation sent to all young adults, unless session relevant only to a specific group (e.g. a women’s only session to discuss menstrual and reproductive health)
The group clinic	Scheduled for afternoon/early evening in usual care setting
	Delivered by group clinic facilitators (diabetes specialist nurse and youth worker) ± an external ‘expert’
	First 15 min: welcome and introductions, ice breaker, setting the scene, ground rules
	Next 60 min (maximum): topic/themed facilitated session, using interactive resources where possible
	Last 15 min: wrap-up to reflect and recap, discuss take-home messages and plan the next group clinic
After the group clinic	Follow-up SMS to all invitees (including those who did not attend) with take-home points, relevant online resources and plans for the next group clinic
	Team (staff) debrief to reflect, learn, and plan the next group clinic

Qualitative analysis followed an iterative, thematic approach moving between inductive and deductive coding and using theory as sensitising device to drive interpretation and dialogue with data (drawing on methodological approaches such as [32, 33]). Field notes (and especially analytical insights contained in them) were used to support reflexive interpretation of data from interviews and group clinics but also as data sources in their own right. Theoretically, we drew on complexity approaches, viewing group clinics as multi-faceted, dynamic change processes, where mechanistic replication and standardization is not sufficient, and attention to uncertainty and ongoing tensions becomes important [34–37]. We used a number of principles (see Table 4) derived from previous research to think with complexity during fieldwork and analysis, particularly in relation to understanding local sense-making, self-organisation, unpredictability, and process interdependencies. These principles derive from literature consolidating learning from complexity theory, i.e. Lanham et al.’s complexity-informed approach to study variation in spread and scale-up of healthcare interventions [37], extended by Greenhalgh and Papoutsi to place more emphasis on human aspects of change [35]. Alongside reflexive analysis of complexity, in this paper, we also present aspects of our qualitative data descriptively to introduce group clinics and contextualise some of the quantitative findings. We used NVivo 11 to support data management.

Sociodemographic, clinical, and service use data were collected across all study sites using questionnaires and standard templates. Participants were also asked to complete the Patient Enablement Instrument (PEI) and Problem Areas in Diabetes (PAiD) questionnaire [38, 39]. In this paper, we present our analysis of participant baseline characteristics by attendance group and site. Comparisons between attendees and non-attendees in implementation sites used *t*-tests for continuous variables and chi-squared for categorical variables. *P*-values < 0.05 were considered statistically significant. We also analysed trajectories of clinical and enablement measures at baseline and 1 year in control and implementation sites to determine future trial feasibility and design, as presented in the detailed study report [30].

Economic evaluation followed the NICE 2013 technology appraisal guide [40]. Using a micro-costing approach, group consultation costs to the NHS were estimated based on Personal Social Services Research Unit (PSSRU) 2018 costs [41]. These included costs of staff running the clinics, arranging appointments and chasing non-attendants, booking rooms and refreshments, and writing in patient notes. Data on staff and resources were collected prospectively using bespoke questionnaires. The average cost per participant was derived by dividing the total cost of running clinics by the number of attendees at each site. Healthcare utilisation data (planned and unplanned contact with diabetes clinicians, general practice, A&E attendances and hospital admissions) were extracted from clinical records for a 12-month pre-intervention period. The cost of usual care was estimated using the National Schedule of Reference Costs 2017-2018 [42]. Individual-level data were combined with unit costs to calculate the total cost of health services use for each participant. Data analyses were conducted in Microsoft Excel 2016.

The study received ethics approval from the Office for Research Ethics Committees Northern Ireland (17/NI/0019). A multi-stakeholder steering group including PPI members provided oversight. More details are included in the final study report for the National Institute for Health and Care Research (NIHR) [30].

## Results

### Developing good value and life-stage oriented care

Introducing group consultations to the diabetes service in the two hospital implementation sites was not straightforward and required careful local experimentation. The appropriate balance between clinical and educational content became a matter of debate, as clinicians were concerned that the typical group consultations format (with standard one-to-one consultations in a group context) would alienate young people who already had low levels of engagement. Instead, they opted for a flexible approach primarily prioritising group interaction on clinically relevant topics, with individual needs addressed indirectly as part of group discussion, rather than replicating one-to-one consultations in the group setting. This also meant that group consultations were not implemented as a substitute to individual clinics but were used purposefully to augment and re-distribute care; when discussing with peers, young people opened up more than they would with clinicians, which resulted in better recognition of their needs and changed the focus of subsequent one-to-one consultations:

‘…they kind of open up. And they may for the first time accept that they've not been taking insulin as recommended, or as advised’ (Interview 25 Diabetes consultant)

Experienced diabetes specialist nurses and other members of the implementation team worked closely with a youth worker, whose contribution was instrumental to developing age- and life-stage appropriate, relationship-based group consultations. The youth worker participated actively in sessions, for example delivering ‘icebreakers’ as a group formation activity and contributing to discussions in a way that would level power dynamics, signifying that group consultations focused on young people’s priorities, rather than purely meeting service or cost-efficiency targets. Clinicians valued youth worker support which allowed them to focus on clinical management without juggling multiple roles for which (in many cases) they had never been trained for (such as facilitating groups of young people).

A typical clinic would start with introductions and an ice-breaker, followed by setting ground rules (see Table 2). Depending on the focus of the session, one or more specialists would join, such as diabetes consultants, dietitians, or psychologists. Topics included healthy eating, blood glucose sensors and measurements, exercise, psychology, sex and healthy relationships, hypos and blood tests, diabetic eye screening and annual review information sessions, sex and healthy relationships, and women’s health, among others.

Although clinicians originally intended for young people to be allocated to specific groups meeting repeatedly and developing long-term relationships throughout the programme, in practice, this proved difficult to sustain and group composition became more fluid. Regular attendees particularly welcomed new participants joining the clinics so they could keep learning from different experiences, but groups also benefited from a certain level of consistency to increase connections between members. The youth worker helped in building affinity quickly between young people who had never met each other so they would open up in discussions and feel supported. At the end of each clinic, participants provided feedback and suggestions for improvement in sessions facilitated by the researcher or the youth worker after clinicians had left the room; this was important for ongoing service co-production (alongside dedicated co-design sessions described elsewhere [30]) to continue meeting patient needs and providing young people with a sense of ownership over this new model of care.

### Key challenges in the implementation and delivery of group consultations: staff experiences

Delivering group clinics involved working with uncertainty and managing multiple interdependencies across diabetes care pathways. It was not simply a matter of providing individual care to multiple people at the same time. Group consultations required a different degree, mode and depth of preparation, and engagement by clinicians and young people alike. The transition was gradual and required changes in established practices but also surfaced and challenged deeply embedded ways of thinking about patient-centred care provision.

Table 4 provides examples of how complexity underpinned the work required to deliver group consultations, including the challenges staff encountered. There was little scope for standardising the processes followed, especially at the beginning, when diabetes specialist nurses were learning through trial and error. Yet, the need to manage uncertainty continued throughout the programme; each session had to be treated as unique and required comprehensive preparation to meet changing patient needs and address all eventualities (unpredictable participation, parents attending, etc.).

**Table 4:** Complexity principles underpinning group clinic delivery, including examples from the study and supporting quotes

Complexity principles [35, 37]	Examples in the study	Relevant data
Acknowledging **uncertainty** and **unpredictability** (e.g. through discovery, learning, and adaptation for multiple plausible futures)	A. Clinicians moved away from a formal, structured mode of delivery introduced at the beginning (e.g. set groups, formal letters) to embrace uncertainty and unpredictability through a ‘trial and error’ approach B. Attendance in group clinics was unpredictable; facilitators initially found this unnerving but eventually acknowledged it as the norm and prepared for all eventualities	*Q1: […] initially we started off with the groups, and we said we will stick to the same group, because we wanted to see how the groups evolved. And within one to two sessions I knew it was not going to work (Interview 1 Diabetes Specialist Nurse)* *Q2: [...] people from first group couldn’t make that particular session, they wanted to join the second or third [group] and then keeping the register going and keeping a tab on who [attended] and when that was quite messy. (Interview 2 Diabetes Specialist Nurse)*
Recognising **self-organisation** (e.g. local patterns of organising and implementation)	C. Established clinical and administrative patterns of organising for individual appointments in each setting hindered group clinic set-up and delivery (e.g. appointment systems, clinic times)—self-organisation was needed to overcome standard operational systems and to co-ordinate between clinicians D. Group consultations were largely driven by participant input therefore relied heavily on patient self-organisation during clinics as well	*Q3: […] just the booking process has created such a problem and how we created the list [for group clinics] because there’s just no infrastructure within the Trust to do group clinics. (Interview 18 Diabetes consultant)* *Q4: […] it was the third session that I did that worked really well where we had the girls and they split up into separate groups and they planned their meals so they spoke about what they, within themselves, currently eat and then came up with ways that they can make it a bit better (Interview 3 Dietician)*
Facilitating **interdependencies** (e.g. supporting effective and fostering new interdependencies)	E. Group sessions had to link with and feed into other care processes, such as one-to-one consultations and group education, and these interdependencies were often difficult to manage F. To customise group clinics, diabetes specialist nurses required in-depth understanding of young people’s needs, acquired through their own experience and through discussions with other clinicians delivering one-to-one care	*Q5: […] when they’re attending my appointment I found that in that group session nobody had looked at their Libre readings and actually highlighted the problems, talked about what changes they needed to make to their insulin on the pump. (Interview 18 Diabetes Consultant)* *Q6: you really need to understand what are their interests, what are their troubles, to actually win their confidence […] in the beginning I spent a lot of time going through learning about each one of them, their history. (Interview 20, Diabetes Specialist Nurse)*
Encouraging and accommodating **sense-making** (e.g. exchanging viewpoints and enabling ongoing, collective reflection)	G. A number of sense-making opportunities were needed to articulate and exchange viewpoints on what this model of care could best contribute H. As a novel concept, group clinics became subject to much negotiation, including on the balance between clinical and educational content	*Q7: When they know that other people are also going through the same problems [in group clinics], then it starts making them feel less guilty. And then that in turn will facilitate how they have the [one-to-one] conversation with their clinicians. (Interview 25 Diabetes consultant)* *Q8: […] in the group clinic if there was eight people there and the nurses had to adjust the insulin for each person […] either everybody around would have found the readings interesting could have learned from that experience or they would have got completely bored. (Interview 18 Diabetes Consultant)*
Developing **adaptive capability** (e.g. tinkering effectively with processes and making judgements)	I. Given group clinic delivery was not a standardised process, staff had to work creatively and flexibly to bring together young people, draw on existing expertise (clinical or other) within and outside the clinic, and sustain value for those involved (patients and clinicians alike)	*Q9: if [speakers] are doing a presentation, I would ask them to send the presentation to me and I will look through it and make sure it's something that would benefit the participants […] you don't want to get them sort of bored by anything, you want to keep them sort of alive and asking questions. (Interview 26 Diabetes Specialist Nurse)*
Attending to human **relationships** (e.g. working together to solve emergent problems)	J. Relationships were at the core of this new care model, both between clinicians to co-ordinate care for young people, between clinicians and patients, but also between patients as they were caring for each other in groups. This needed careful consideration of boundaries and interaction dynamics and required attention and time commitment	*Q10: […] relationships take time to build, specially trusting relationships. […] And it’s not just me building relationships with the young adults, it’s me building relationships with the clinicians. (Interview 1 Diabetes Specialist Nurse)* *Q11: […] it has been a quite rewarding and challenging experience for me, as well. So, every time I say something, I'm questioning myself again internally. So, is that too much for them? Is that too little for them? Or is it reaching everyone? (Interview 25 Diabetes Consultant)*
Harnessing **conflict** productively (e.g. viewing conflicting perspectives as raw ingredients for multifaceted solutions)	K. Micro-conflicts emerged both among clinicians on how best to deliver group clinics and between young people on how best to self-care	*Q12: […] in some of the sessions there’s been a bit of a conflict, and conflict management can be quite tricky. And I certainly know that the nurse was quite upset about it after that and so that needs a little bit of help. (Interview 18 Diabetes consultant)*

Self-organisation underpinned efforts to informally co-ordinate between different clinicians providing one-to-one and group care to young people, in terms of selecting participants for group clinics, understanding their needs, inviting the right experts to contribute, and managing interdependencies with other care processes (e.g. diabetes education, individual appointments) (Q3). In-depth clinical and relational knowledge about young people mattered when deciding how to bring them together and facilitate the sessions so they would benefit most; this knowledge needed to be collectively accumulated and negotiated between different clinicians involved and drawn out of medical records. Informal, improvised, and spontaneous interactions between clinicians enabled ongoing co-ordination, largely driven by the efforts of the diabetes specialist nurses, but also other staff involved (e.g. diabetes consultants, research officer). Other practical and logistical challenges ensued, such as securing seminar rooms, adjusting booking processes, and maintaining continuity with the rest of the diabetes service (Q5).

Formal and informal opportunities were needed for reflection and sense-making, and to support learning within and across implementation sites (e.g. implementation and project meetings, co-design, training sessions). Development of adaptive capability became important for clinicians who were delivering a new model of care highly dependent on human relationships. Group clinics involved the dual challenge of delivering good clinical care and education, while facilitating a group of young people. In some cases, it was important for clinicians to engage in emotional work to support groups where conflict and competition emerged and to ensure outcomes remained positive (Q12). Health professionals drew on their skills consulting with young people, but also attended group facilitation training, held regular debriefs between implementation and clinical teams for ongoing adjustment of the model, and derived significant learning from on-the-job trial and error.

### Attendance and young people’s motivations

Despite significant effort, mean attendance was relatively low at 32% for site A and 33% for site B—a challenge already familiar to those delivering young adult services. Local teams had to work creatively to make sessions worthwhile regardless of how many young people ended up attending. Despite suggestions that a ‘good’ session should include 6–8 patients, in practice, the ‘right’ number largely depended on the focus and facilitation mode of each session (e.g. more young people could meaningfully participate in a session about exercise compared to psychology). Larger groups did not always guarantee high levels of contribution; there were successful groups with as many as 4 young people who identified with each other and shared openly.

[…] it seemed to be around sort of three, four, five we were getting [to attend], even though you know, we invited more than twenty patients, within a good amount of time. So I think just trying to make sure a lot of people, or as many people as possible would attend, was the biggest challenge. (Interview 29, Diabetes Specialist Nurse)

An average of 4–5 young people attended each group consultation at both sites. Higher attendance rates were recorded when a small group of selected young people were invited for a specific care-focused intervention, such as flash glucose monitoring follow-up (range of 83–100% in three sessions). Variable attendance rates were observed at broader educational and self-management sessions (e.g. psychological health, healthy eating), especially when there was an open invite to all young people recruited at each site (range of 0–60% in 25 sessions). As group clinics continued, attendance was mostly from those who had attended previous sessions, suggesting group consultations appealed to and continued to attract a specific set of young people (5–6 young people attended 5–10 sessions in site A and 3–4 in site B), but the majority only attended a small number of sessions.

Some young people expressed feeling motivated to participate in group consultations, mainly to meet others with diabetes in their age group. However, others were unable to fit group consultations alongside standard, individual diabetes care and other responsibilities (such as family, education, employment, social life). They also expressed feeling ambivalent or in ‘two minds’ about this new service model as they did not know what to expect or did not feel ready to engage with their condition; some overcame initial fears although others chose not to participate at all.

But yeah, it’s like having a group clinic is so much nicer, in order to meet people. But then on the other hand, I think because you don’t really know them, you don’t have that personal connection with them, you don’t really want to voice out everything that you’re going through. Do you get that? I’m a quiet person, like I wouldn’t tell people what I’m going through if I don’t really know them. So I was in like two minds. (Interview 12, Patient 7—never attended)

If I’m being honest, at the beginning, I didn’t want to come. I did, but I didn’t. I just like - oh, when is it going to be, is it going to be really long, I might not like it. But I still came. And I liked it. I was like ‘okay, this isn’t what I was expecting’. I was not expecting it to be so laid back. I don’t know. It was really comfortable, the setting. (Interview 10, Patient 5—regular attendee).

Not all young people had disclosed their diabetes in their communities and they were unsure how to share deeply personal experiences. There was also an underlying resistance to supporting a new consultation mode if this would mean reducing individual appointments for cost efficiency.

### Differences between attenders and non-attenders in implementation sites

In Tables 5 and 6, we present baseline characteristics of the 73 young people recruited in the two implementation settings, comparing those who attended one or more group clinics to those who did not attend any group clinics at each site (further comparisons with participants recruited in control sites are available in the detailed project report [30]).

**Table 5:** Participant baseline characteristics by attendance group and site

	Participants	Age (years)	Age (years) at diagnosis	Male	Ethnic minority	In education	Employed	Living in highest deprivation quintile	English as first language	Type 1 diabetes	Technology use* in the last year	Previous group education
	***n***	Mean (SD)	Mean (SD)	***n*** (%)	***n*** (%)	***n*** (%)	***n*** (%)	***n*** (%)	***n*** (%)	***n*** (%)	***n*** (%)	***n*** (%)
**Site A (All)**	**50**	**21 (2.7)**	**14 (6.9)**	**24 (48)**	**40 (80)**	**13 (26)**	**15 (30)**	**26 (52)**	**22 (44)**	**40 (80)**	**17 (34)**	**11 (22)**
**Site A attenders**	**23**	21 (2.7)	11^a^ (6.9)	11 (48)	20 (87)	7 (30)	9 (39)	11 (48)	9 (39)	20 (87)	10 (44)	9 (39)^b^
**Site A non- attenders**	**27**	21 (2.8)	16^a^ (6.2)	13 (48)	20 (74)	6 (22)	6 (22)	15 (56)	13 (48)	20 (74)	7 (26)	2 (7) ^b^
**Site B (All)**	**23**	**20 (2.3)**	**11 (6.1)**	**10 (43)**	**18 (78)**	**9 (39)**	**10 (23)**	**4 (17)**	**15 (65)**	**19 (82)**	**5 (22)**	**7 (30)**
**Site B attenders**	**14**	20 (2.3)	11 (4.8)	6 (43)	10 (71)	4 (29)	7 (50)	3 (21)	9 (64)	12 (86)	3 (21)	2 (14)
**Site B non- attenders**	**9**	21 (2.1)	11 (8.1)	4 (44)	8 (89)	5 (56)	3 (33)	1 (11)	6 (67)	7 (78)	2 (22)	5 (56)

Comparisons between attendees and non-attendees at sites A and B use *t*-tests for continuous variables and chi-squared for categorical variables

*Technology use refers to the use of newer diabetes technologies, including flash glucose monitoring, continuous glucose monitoring, or insulin pump therapy. *p* values ≥ 0.05, except ^a^*p* value = 0.033 when comparing age at diagnosis between participants who attended vs. non-attended clinics at site A and ^b^*p* value = 0.053 when attendees vs. non-attendees at site A were compared

**Table 6:** Baseline clinical characteristics and questionnaire scores by attendance group and site

	Number of participants	HbA1C	Frequency of blood glucose testing per day	PAID score	PEI Score	Planned diabetes appointments attended	Emergency department attendances (diabetes related)	Inpatient diabetes-related admissions	Primary care diabetes-related consultations
	***n***	Mean (mmol/mol)	Mean	Mean	Mean	***n*** (%)	Mean	Mean	Mean
**Site A (All)**	**50**	**73**	**2**	**25**	**7**	**70**	**0.3**	**0.2**	**2**
**Site A attenders**	**23**	73	3	23	8	70	0.2	0.1	1
**Site A non-attenders**	**27**	72	2	27	7	70	0.3	0.2	2
**Site B (All)**	**23**	**80**	**3**	**27**	**6**	**70**	**0**	**0**	**0.6**
**Site B attenders**	**14**	68^a^	3	29	6	80^b^	*-*	*-*	0.5
**Site B non-attenders**	**9**	98^a^	3	25	7	50^b^	*-*	*-*	0.7

Comparisons between attendees and non-attendees at sites A and B use *t*-tests for continuous variables and chi-squared for categorical variables. All *p* values ≥ 0.05, except ^a^*p* value 0.023 in comparison of baseline HbA1c between attendees and non-attendees at site B and ^b^*p* value = 0.009 in comparison of planned diabetes appointments attended in the 1 year prior to joining the study between participants attending or not attending group clinics. ‘–’ denotes insufficient data points for analysis

At site A, comparing participants who did (*N* = 23) and did not (*N* = 27) attend any group clinics, there were no significant differences in sex, ethnicity, deprivation, speaking English as a first language, type of diabetes, or use of technology within the last year (Table 5). Those who attended were on average diagnosed at a younger age (11 vs. 16 years) and more likely to have attended group education sessions in the past (39% vs 7%), with borderline statistical significance (*p* = 0.033 and 0.053 respectively). There were no statistically significant differences in these variables when comparing attenders (*n* = 14) and non-attenders (*n* = 9) at site B.

Comparison of attenders and non-attenders at site A showed no statistically significant differences between these groups when comparing baseline clinical characteristics and questionnaire scores (Table 6). In contrast, attenders at site B had better glycaemic control (mean HbA1C 68 vs. 98 mmol/mol, *p* = 0.023) and had attended 80 vs. 50% of planned appointments within the previous year (*p* = 0.009).

### Young people’s experiences in group clinics

Young people who attended group clinics (especially repeat attenders) discussed their experiences as predominantly positive: they felt better understood and supported, learnt new things from peers and clinicians, and were better able to normalise diabetes self-care. Only in a few instances did young patients express (initial) reluctance to share clinical details or found peer comparison challenging; in these cases, internal dynamics required careful management by clinicians.

Group clinics provided the opportunity to discuss emotions and frustrations with others going through similar challenges. Young people found peers could understand and identify with their experiences, which made them feel less isolated. They felt better able to engage in open discussion as they gained encouragement from each other when they started to realise how all were struggling to follow clinical recommendations:

F1: How, I just want to ask generally, how are you guys, like those on type 1, how are you guys finding carb counting? How do you get round it, how do you start all up? F2: I’m not going to lie I haven’t been really carb counting. F1: OK I’m glad to [have asked], I mean it’s a bad thing but it’s like I’ve been struggling so much I’m just like I’ve given up with it totally. Are you the same like? F2: (indicates agreement) (Site A, exchange between female patients in Clinic 2)

Being able to explore emotional challenges of living with diabetes was repeatedly mentioned as a key aspect of positive experiences in group clinics, compared to individual appointments, where young people expressed reluctance to voice their difficulties:

The one-to-one is more personalised, scientific. […] Where [the group clinic] is more lifestyle based. It’s more about how to live with your diabetes, rather than just manage it […] With the doctor, I kind of want to just get it over and done with really quickly, and then just go. So I wouldn’t, I don’t try to ask as many questions or I just forget. (Interview 27, Patient 16)

One of the young people with type 2 diabetes did express feeling alienated initially, in a clinic where everyone else had type 1 diabetes, but then explained: ‘it was [a] very welcome [environment] so, feelings of being left out didn’t last too long to be honest’ (Interview 24, Patient 15)

Another participant suggested that they felt less comfortable with individual appointments because they perceived them as ‘professional’—which at their life stage seemed alienating, as they were unsure how to navigate the rules of engagement and match them with their own priorities.

Social and situated learning emerged through a combination of patient input and clinical advice (e.g. on alternating injection sites, ketone testing or avoiding hypos), carefully facilitated by the diabetes specialist nurses who ensured young people gained insight without feeling judged or criticised. Learning emerged both for those newly diagnosed and for those diagnosed at a younger age, who had been looked after by their families and were only just beginning to learn how to care for themselves independently. Clinicians were surprised that young people had not already acquired this learning through individual appointments on similar topics.

Patient participants talked about how group discussions with peers helped them think about their diabetes differently and normalise their experiences through getting to know how others approached their self-care. This even resulted in some feeling more confident and comfortable with their condition to the extent they started disclosing to their workplace and friends:

[…] within the workplace I would never tell people that I’ve got diabetes, and stuff like that. Now, the other day I was speaking to my friend about where I should be injecting, where I shouldn’t be injecting. Feel like now I’m a bit more confident and comfortable with it. (Interview 13, Patient 8)

There was, however, some reluctance to share clinical details considered private (e.g. glucose levels) or have test results displayed on the computer screen for discussion. Others were not always prepared to discuss self-care aspects they were struggling with or to manage a group discussion that might have led to sharing beyond what they were comfortable with, so chose to control their contributions. For those newly diagnosed, comparison with peers was not always motivating, especially when they were comparing themselves with others doing worse:

And so what I was thinking is that would it get to a stage where it’s going to be hard for me to manage my diabetes. Yeah, it definitely did freak me out a bit, yeah. (Interview 14, Patient 9)

### Costs of group consultations and health care

The average staff costs for setting-up and delivering group consultations were similar across the two implementation sites (£572 for site A and £545 for site B) (Additional file 1: Tables 1a and 1b). The average cost of clinic per participant was marginally higher in site A (£158) compared to site B (£127), due to poorer attendance in the former (average number of participants was 3.7 for site A versus 4.5 for site B) (Additional file 1: Table 2). The study participants attended on average 3.6 out of 5.9 scheduled appointments per year, including consultations with a diabetes doctor, diabetes specialist nurse, dietician, and psychologist. The average annual cost of scheduled care was £723 per patient per year. The study participants had on average 3.9 unscheduled contacts per year including A&E visits, hospital admissions, and contacts with general practitioners and diabetes specialist nurses. The average annual cost of unscheduled care was £2566 per patient (Additional file 1: Table 3).

## Discussion

Our study shows how improving quality of diabetes care through group consultations means, first and foremost, paying attention to complexity and adaptive capability. A standardised, one-size-fits-all approach would be difficult to sustain when implementation teams have to work sensitively across multiple, complex interdependencies in the context of standard diabetes care and education. Clinicians in our study maintained value in group consultations by: closely and flexibly following young people’s needs (clinical and educational) and engagement styles; aligning with local service priorities and achieving co-ordination despite standard operational systems and service limitations; building and sustaining relationships among clinicians and young people; facilitating emotional connections for social and situated learning (for young people and staff alike); and enabling a safe environment for patients to feel supported and normalise diabetes experiences. There were, however, instances where peer comparison did not lead to self-care motivation, and group dynamics had to be managed carefully.

Patient attendance was one of the most challenging aspects of the programme. A core group (about one third of invitees) participated with enthusiasm, but others were unsure what to expect or what sharing in a group setting would entail. Similar attendance challenges have been identified in previous work. In their systematic review on non-attendance in standard diabetes outpatient consultations, Brewster et al. found non-attendance rates generally ranging between 10 and 30%, with one study reporting non-attendance at 76% [11]. Previous qualitative studies on group consultations tend to involve patients selected as more ‘suitable’ to increase engagement rather than assess broader acceptability [43]. A more flexible approach to attendance and engagement suited young people in our programme, but placed greater responsibility on staff to create in-the-moment affinity within groups, and remain sensitive to group dynamics and changing patient needs.

Operational alignment and good facilitation skills have been highlighted as enablers in research on group consultations [22]. For example, a recent qualitative study in UK general practice emphasised the need for facilitators to have a broad set of skills in health coaching, behaviour change, but also organisational and presentation skills [26]. In our group consultations for young people, the youth worker role was instrumental in supporting engagement and providing additional capacity. An international consensus conference aiming to improve outcomes for young people with type 1 diabetes also concluded that youth worker involvement in diabetes care and transition is crucial [44]. Given group consultations were critically dependent on key staff (youth worker, diabetes specialist nurse) with advanced skills in co-ordination and facilitation (beyond common practice in standard care settings, and with additional research resources), there are questions as to how scalable and sustainable this model of care might be.

Although sessions largely followed a standard format (see Table 2), their core focus shifted depending on patient-led priorities, clinical assessment, group engagement, and other pragmatic considerations (equipment, resources, etc.). To enable familiarisation with group-based care, initial sessions were geared more towards educational topics and self-management (seen as less threatening), instead of clinical care and blood test discussions which clinicians thought may further alienate young people. This was in contrast to how group consultations are typically conceptualised in the literature (primarily as involving clinical care, e.g. in the context of annual diabetes reviews as we found in recent work in English general practice [24]) but necessary to facilitate implementation for a young age group. The balance shifted towards clinical care for a core group of repeat attenders, especially those using Libre blood glucose sensors, but without reaching a point where group consultations could incorporate enough clinical content to replace individual appointments. Instead, over the 2-year implementation period, an understanding emerged within clinical teams that group consultations were best placed to augment and support individual diabetes care for young people, fulfilling different, but synergistic, purposes towards high-quality diabetes care.

By observing young people interacting with each other in group consultations, clinicians understood more about their circumstances, challenges, and engagement preferences, which created better potential for patient support in individual consultations (previously characterised by avoidance and mistrust). Debating what makes a ‘good’ group consultation was a core part of the study and resulted in a fluid approach carefully balancing clinical care, peer support, and education and guided by co-production and clinical judgement. However, when delivering group clinics at scale, this context-specific tailoring may be difficult to achieve and will require additional investment to develop a blended model that works for each specific setting and patient population.

Baseline data showed few differences between those attending and not attending group clinics in the implementation sites. In site A, young people with poor diabetes control and engagement were just as likely to attend group clinics, as those already well engaged in their care, whereas in site B, we found those attending to have better glycaemic control and service engagement compared to those not attending. Yet, there were barriers to obtaining quantitative data, including the high turnover of young people with diabetes within the clinics studied and difficulties in accessing clinical systems for data collection, which limited our ability to draw conclusions from statistical analysis.

The cost-effectiveness of group consultations is yet to be established. Results from this study suggest that the annual cost of unscheduled care for young people with diabetes is almost three times higher compared to the cost of scheduled care. The main cost drivers were hospitalisations and face-to-face consultations with general practitioners. Group consultations could be good value for money if they prevent at least one visit to A&E (£297) or to a general practitioner per year (£211) [45].

Further research is needed, at scale, to evaluate a combined model of individual and group-based clinical care against clinical and service outcomes and cost-effectiveness including through the use of routinely collected quantitative data (e.g. National Diabetes Audit, Hospital Episode Statistics) to facilitate the data collection process.

More work is needed to better understand how to bring together one-to-one diabetes care, structured education, and group consultations to respond to emergent clinical and self-care needs for different patients at different points in time. We found ongoing and reflexive co-production in the context of service delivery to play a significant role in co-ordinating different aspects of diabetes care, but this required resources, effort, and capacity beyond what is usually available in clinical settings. Group consultations cannot be seen as a cost-cutting measure as they require dedicated staff with non-traditional skills in group facilitation and adaptive capability to deliver patient-focused and locally integrated care. There is also potential to explore remote, digital options in group-based care (e.g. see [24]) which could play a role in supporting continued engagement for young people.

### Strengths and limitations

This study combined qualitative and quantitative methods (underpinned by previous evidence synthesis [22]) to assess patient and staff experiences, potential for measurement of clinical and service outcomes, and costs. Group consultations engaged young adults in more ethnically diverse and socially deprived areas than UK averages. The ‘researcher-in-residence’ spent considerable time with clinical teams over the 2-year implementation period and used multiple qualitative methods to develop in-depth understanding. Yet, we may not have adequately captured views of those who refused to take part in group consultations or had negative experiences. Although the ‘researcher-in-residence’ worked closely with clinical teams (without being physically co-located in the hospitals full time), capacity to support implementation through translation of research evidence was often limited due to pragmatic considerations and resource constraints on the front-line. There were sometimes tensions between, on the one hand, creating relationships with clinical teams and, on the other, sustaining ethnographic and critical distance, with the researcher also becoming acutely aware of the challenges working across research and practice boundaries. Our theoretical orientation to complexity enabled us to surface multiple interacting challenges in relation to planning and delivering group consultations. Yet, in this paper, we have not been able to do full justice to the complexities we encountered and are therefore planning further publications. An orientation to complexity also guided us away from providing recommendations for quick ‘fixes’ or simple recipes—instead, we convey our rich learning in the hope that it will be useful for others engaging in group consulting.

Despite significant efforts, we encountered variation in quantitative data completeness and challenges obtaining participant-level data, and the small sample size and low event rate restricted the potential to draw strong quantitative conclusions (although the study was exploratory and not designed to be powered to quantitative clinical outcomes). Our cost analysis only provides early insights into the economics of delivering group-based care.

## Conclusions

There is potential to deliver group consultations in ways that engage and fulfil needs for young people with diabetes in ethnically diverse, socio-economically deprived settings. However, this relies on healthcare staff with capacity, skills, and remit to engage in local tailoring and careful adaptation of this model of care, proactive alignment and integration with service priorities, and sensitive, youth-focused engagement, while working closely with young people as active partners in shaping high-quality care provision.

Additional file 1 provides more details on the resource use and costs associated with running group clinics in the two implementation sites as well as further information on resource use and costs of usual care for scheduled and unscheduled contacts at site B.

## Supplementary Information

**Additional file 1: Table 1a.** Resource use and costs associated with running group clinics, site A. **Table 1b.** Resource use and costs associated with running group clinics, site B. **Table 2.** Resource use and costs of usual care: scheduled contacts, site B. **Table 3.** Resource use and costs of usual care: unscheduled contacts, site B.

## Data Availability

The datasets used and/or analysed during the current study are available from the corresponding author on reasonable request.

## Abbreviations

NICE: National Institute for Health and Care Excellence
NIHR: National Institute for Health and Care Research
PAiD: Problem Areas in Diabetes
PEI: Patient Enablement Instrument
PSSRU: Personal Social Services Research Unit
