# Supplementary material for: Implementation and delivery of group consultations for young people with diabetes in socioeconomically deprived, ethnically diverse settings
Source: BMC Med. 2022 Nov 25;20:459. doi: 10.1186/s12916-022-02654-0 (PMC9701006; doi:10.1186/s12916-022-02654-0)
Supplement: Supplementary file 1 — Additional file 1: Table 1a. Resource use and costs associated with running group clinics, site A. Table 1b. Resource use and costs associated with running group clinics, site B. Table 2. Resource use and costs of usual care: scheduled contacts, site B. Table 3. Resource use and costs of usual care: unscheduled contacts, site B. [file 12916_2022_2654_MOESM1_ESM.doc]

Additional File 1.

Table 1a. Resource use and costs associated with running group clinics, site A.

| **Resource** | **N (average)** | **Range** | **Total cost, £** |
| --- | --- | --- | --- |
| Number of clinics | 6 |  |  |
| Average number of participants invited | 26 | 24-27 |  |
| Average number of participants attended | 4.5 | 3-6 |  |
| Number of staff delivering the clinics |  | 3-4 |  |
| Time of running the clinics, minutes | 67 | 30-95 | 1,590.09 |
| Time of preparing room and materials, minutes | 13 | 5-15 | 56.25 |
| Time of arranging appointments and chasing non-attenders, minutes | 260 | 240-270 | 1,170.00 |
| Time of booking room and confirmation, minutes | 17.5 | 5-30 | 78.75 |
| Time of making patient notes, minutes | 84 | 50-137 | 309.38 |
| Time of other activities, minutes | 51 | 30-82 | 227.81 |
| Other expenses (travel, room hire, printed materials, etc.) |  |  | 0.00 |
| **Total cost of group clinics 3,432.28** | | | |
| **Average cost per clinic 572.05** | | | |
| **Average cost per participant (one clinic) 127.12** | | | |

Table 1b. Resource use and costs associated with running group clinics, site B.

| **Resource** | **N (average)** | **Range** | **Total cost, £** |
| --- | --- | --- | --- |
| Number of clinics | 22 |  |  |
| Average number of participants invited | 17 | 2-42 |  |
| Average number of participants attended | 3.7 | 0-7 |  |
| Number of staff delivering the clinics |  | 2-3 |  |
| Time of running the clinics, minutes | 135 | 120-180 | 8,939.16 |
| Time of preparing room and materials, minutes | 16 |  | 575.00 |
| Time of arranging appointments and chasing non-attenders, minutes | 71 |  | 1,292.00 |
| Time of making patient notes, minutes | 35 |  | 715.00 |
| Time of other activities, minutes | 12 |  | 202.50 |
| Lunch |  |  | 78.00 |
| Other expenses |  |  | 190.00 |
| **Total cost of group clinics 11,991.66** | | | |
| **Average cost per clinic 545.08** | | | |
| **Average cost per participant (one clinic) 157.79** | | | |

Table 2. Resource use and costs of usual care: scheduled contacts, site B.

| **Contact** | **Number of patients with contacts** | **Number of contacts** | **Total cost, £** | **Average cost per patient, £** |
| --- | --- | --- | --- | --- |
| Endocrinologist (planned) | 38 | 101 | 15,554 | 409 |
| Endocrinologist (attended) | 31 | 55 | 8,470 | 273 |
| Diabetes specialist nurse (planned) | 41 | 152 | 10,184 | 248 |
| Diabetes specialist nurse (attended) | 39 | 101 | 6,767 | 174 |
| Dietician (planned) | 25 | 34 | 2,924 | 117 |
| Dietician (attended) | 17 | 21 | 1,806 | 106 |
| Psychologist (planned) | 5 | 6 | 1,020 | 204 |
| Psychologist (attended) | 1 | 1 | 170 | 170 |
| **Total planned appointments** | | **293** | **29,682** | **979** |
| **Total attended appointments** | | **178** | **17,213** | **723** |
| **Total missed appointments** | | **115** | **12,469** | **256** |

Table 3. Resource use and costs of usual care: unscheduled contacts, site B.

| **Contact** | **Number of patients with contacts** | **Number of contacts** | **Total cost, £** | **Average cost per patient, £** |
| --- | --- | --- | --- | --- |
| A&E | 8 | 13 | 1,899 | 237 |
| Hospitalisation | 6 | 8 | 9,118 | 1,520 |
| Diabetes specialist nurse (face to face) | 7 | 19 | 1,273 | 182 |
| Diabetes specialist nurse (non-face to face) | 28 | 76 | 1,938 | 69 |
| GP (face to face) | 22 | 44 | 7,524 | 342 |
| GP (non-face to face) | 2 | 6 | 294 | 147 |
| Practice nurse (face to face) | 16 | 29 | 1,102 | 69 |
| **Total unscheduled contacts** |  | **195** | **23,149** | **2,566** |
